# Lack of Monitoring Is Associated with Risk of Acute Kidney Events among Patients with Inflammatory Bowel Disease

**DOI:** 10.3390/jcm11112954

**Published:** 2022-05-24

**Authors:** Hamza Achit, Laurent Peyrin-Biroulet, Carole Ayav, Francis Guillemin, Luc Frimat

**Affiliations:** 1 Clinical Epidemiology Centre CIC-1433 CHRU-Nancy, Inserm, Université de Lorraine, 54500 Vandoeuvre-les-Nancy, France; c.ayav@chru-nancy.fr (C.A.); francis.guillemin@chru-nancy.fr (F.G.); 2 Inserm NGERE and Department of Gastroenterology, University Hospital of Nancy, Université de Lorraine, 54511 Vandoeuvre-les-Nancy, France; peyrinbiroulet@gmail.com; 3 Department of Nephrology, University Hospital of Nancy, 54511 Vandoeuvre-les-Nancy, France; l.frimat@chru-nancy.fr

**Keywords:** monitoring, IBD, renal involvement

## Abstract

Background: Although the iatrogenic risk of kidney failure is infrequent with treatment for inflammatory bowel disease (IBD), the repercussions for the patient could be major. The aim of this study was to assess the incidence of kidney events in IBD and to examine the protective effect of kidney function monitoring. Methods: In the French National Health Insurance database, 94,363 patients had a diagnosis of IBD between January 2010 and December 2016. By using a survival model with time-dependent covariates, we analyzed the time from inclusion in this IBD cohort to the first hospitalization for acute kidney impairment (AKI) according to patient characteristics, comorbidities, IBD phenotype and presence of monitoring. Results: A total of 693 patients were hospitalized for AKI, with an incidence of 1.36/1000 person–years (95% confidence interval [CI] 1.26–1.47). The incidence of AKI was lower than those without 5-aminosalicylic acid (5-ASA) use. Patients with 5-ASA use rarely had any lack of monitoring as compared with those not under 5-ASA use (3% vs. 17%). On multivariate analysis, lack of monitoring was associated with a substantial risk of AKI (hazard ratio 3.96, 95% CI [3.20–4.90], *p* < 0.0001). Conclusions: Increased frequency of monitoring is essential to identify nephropathy at an early stage and avoid the progression to chronic kidney disease.

## 1. Introduction

The nephrotoxicity of treatments for inflammatory bowel diseases (IBDs) is highly debated, and many studies have attempted to clarify this issue. However, findings can be contradictory and confusing regarding both the frequency of nephrotoxicity and the type of treatments involved [1,2]. Treatment with 5-aminosalicylic acid (5-ASA) is the main focus of studies on kidney impairment in IBD [3,4]. In patients receiving 5-ASA, regular kidney function monitoring is recommended.

Many studies have reported the toxic effects of 5-ASA on the kidney [5,6,7,8]. Recent studies concluded that nephrotoxicity was not associated with 5-ASA use but rather with disease activity and comorbidity [9,10,11,12]. Indeed, independent of the treatment, many extra-intestinal manifestations (e.g., nephrolithiasis, tubulointerstitial nephritis, etc.) occur during the IBD course [13]. One study identified a genetic predisposition to nephrotoxicity in patients receiving 5-ASA [14]. Other studies identified a risk of nephrotoxicity for patients with Crohn’s disease but not ulcerative colitis [15,16]. 

Regardless of the causal factors, the unpredictable character of renal impairment suggests that IBD patients should be carefully monitored. Any delay in kidney diagnosis could lead to irreversible damage due to the silent and asymptomatic nature of the impairment. The British Society of Gastroenterology guidelines advises regular function monitoring for patients under 5-ASA treatment [17]. Regardless, practices remain heterogeneous across practitioners and countries. In a UK study, researchers analyzing a local database of 612 IBD patients found that up to 48% did not have renal function measurement during treatment. In addition, a number of patients started 5-ASA treatment even though baseline renal function testing identified chronic kidney disease [18]. These results differ greatly from those of a French questionnaire-based study in which most IBD patients stated that they were well informed and adherent to kidney function monitoring with a 5-ASA prescription [19]. 

The aim of this study using data for a nationwide IBD cohort was to estimate the risk of AKI in IBD and to examine the impact of renal function monitoring on preventing associated renal events.

## 2. Materials and Methods

### 2.1. Data Sources

This study was based on the French SNDS owned and managed by the National Sickness Insurance Fund (*Caisse Nationale d’Assurance Maladie* [CNAM]). This latter organization ensures the universal coverage of healthcare expenses for about 99% of the 67 million French citizens from birth (or immigration) to death (or emigration). Thus, SNDS is among one of the world’s largest continuous, homogenous claims databases. By using a unique anonymous identifier, the SNDS merges information for reimbursed claims from (1) the French national hospital discharge database (*Programme de médicalisation des systèmes d’information*), which since 2006 has provided individual medical information on all hospital admissions in France, including discharge diagnoses (International Classification of Diseases, 10th revision [ICD-10]) and medical procedures performed; (2) the national healthcare system claims database (SNIIRAM), which contains outpatient data (date and type of reimbursed drug dispensation and number of units, attribution of long-term chronic disease coverage [*affection de longue durée* (ALD)], date and nature of paramedical interventions and laboratory tests, date of birth, sex, and date of death); and (3) the national cause-of-death registry (CépiDc). This interconnection of medical databases allows for constituting a cohort of patients with specific diseases based on many tracking indicators (e.g., illness-specific drug use, chronic illness recognized and fully exempt from co-payment by the CNAM). In addition to medical information, many socio-demographic characteristics including sex, date of birth and residence area are available. 

This study was approved by the French Data Protection Authority (*Commission Nationale de l’Informatique et des Libertés*). All data used in this study contained only de-identified patient records.

### 2.2. Study Population

All patients newly exempt from co-payment by the CNAM because of IBD between 1 January 2010 and 31 December 2016 were included in the study. IBDs consist of 30 chronic illnesses fully covered by CNAM. Exemption status was used rather than other indicators of IBD morbidity (e.g., IBD drug use, hospitalization for IBD) because it allows for confirming the accuracy of the IBD diagnosis. Indeed, the disease is carefully diagnosed before patients are admitted to a co-payment exemption regimen. Analysis was restricted to patients with no history of kidney impairment before inclusion in the IBD cohort. Therefore, we excluded all patients hospitalized for kidney impairment before the date of IBD diagnosis. 

### 2.3. Statistical Approach

The incidence of AKI was estimated by dividing the number of events by 1000 person–years of follow-up for each category of patients. In the multivariate analysis, time from inclusion in the IBD cohort to the first hospitalization for kidney impairment was analyzed according to patient characteristics and risk factors including age, sex, IBD phenotype (Crohn’s disease/ulcerative colitis), comorbidities and presence of monitoring. A Cox proportional-hazards model was used after adjusting for baseline and time-dependent covariates as follows:

Log h_i_(t) = *α*(t) + *β*_1_X_i1_ + *β*_2_X_i2_(t)

where for patient “i”, X_1_ is fixed and X_2_ is a time-varying covariate. Hazard ratios (HRs) and 95% confidence intervals (CIs) were estimated. Results from the stepwise procedure were retained. Analyses were performed with SAS Enterprise Guide 7.1 (SAS Institute Inc., Cary, NC, USA). *p* < 0.05 was considered statistically significant.

### 2.4. Screening for “Silent” Kidney Damage

Screening is essential to prevent serious complications. Our hypothesis was that more regular monitoring is associated with decreased risk of severe kidney complications. Serum creatinine and proteinuria are the main tests of kidney function monitoring. These tests are recommended by the expert group *Groupe d’Etude Thérapeutique des Affections Inflammatoires du Tube Digestif* and are the most commonly performed in daily practice.

### 2.5. Comorbidities

Comorbidities known to be associated with increased risk of kidney impairment in the general population include obesity, diabetes, coronary artery diseases, recent stroke, dyslipidemia, arterial disease, hypertension and heart failure. The diagnostic codes for these comorbidities in the SNDS database are in Appendix A. 

### 2.6. Outcomes

The main outcome was patient hospitalization related to AKI. The occurrence of this event was identified in the SNDS database by using the ICD-10 codes. Only codes for AKIs were retained: N00, N01, N04, N10, N141, N142, N144, N17. More details on ICD codes are in Appendix B. The date of the first hospitalization was retained if many hospital stays were recorded.

## 3. Results

### 3.1. Baseline Characteristics of the Study Population 

Between 1 January 2010 and 31 December 2016, 94,363 patients in the French population were recorded as exempt from co-payment by the CNAM because of IBD; patients were followed up to 31 December 2017 for an average follow-up time of 5.38 (2.05) years. The mean (SD) age at IBD diagnosis was 37.2 (17.2) years. 

### 3.2. Incidence of AKI According to Patient Characteristics and Comorbidities

We observed 693 cases of hospitalization for AKI as the main diagnosis during the follow-up. The incidence was 1.36 per 1000 person–years [95% CI 1.26–1.47]. The mean and median follow-up time for hospitalized patients since IBD onset was estimated at 2.95 (2.07) and 2.53 years, respectively.

The AKI group included predominantly women, patients with Crohn’s disease and older patients than those without AKI (Table 1). At IBD diagnosis, the mean age of hospitalized and non-hospitalized patients was 43.3 (20.8) and 37.2 (17.2) years. The prevalence of comorbidities was higher for patients with than without AKI.

On multivariate analysis, many comorbidities were associated with an increased risk of AKI (Table 2). Most corresponded to diseases known as risk factors in the general population. For IBD patients, the risk of kidney failure was associated with diabetes, recent stroke, dyslipidemia, arterial disease, heart failure and hypertension.

### 3.3. Incidence of AKI According to Monitoring

We found a significant association between AKI and lack of monitoring in the IBD cohort. The risk of AKI was associated with no monitoring as compared with monitoring (HR 3.7, 95% CI [3.0–4.6], *p* < 0.0001) (Table 3) and remained associated with multivariate analysis after adjustment (HR 3.96, 95% CI [3.20–4.90], *p* < 0.0001) (Table 2). 

### 3.4. Incidence of AKI According to ASA Use

The AKI incidence was significantly lower for patients receiving 5-ASA as compared with those without 5-ASA (1.04 vs. 2.27 per 1000 person–years) (Table 3). However, on bivariate analysis, lack of monitoring was rare for patients receiving 5-ASA: 3% versus 17% in the group not receiving 5-ASA. On multivariate analysis, for the general IBD population, for IBD patients receiving 5-ASA, the lack of monitoring was associated with a higher risk of AKI (HR 2.36, 95% CI [1.51–3.68], *p* = 0.0002) (Table 2).

## 4. Discussion

By using a French nationwide database, we estimated the incidence of AKI inducing a hospital stay in a representative cohort of IBD patients; the incidence was 1.36 per 1000 person–years (95% CI 1.26–1.47). The incidence of all kidney events could be much higher in terms of all events diagnosed at an early stage after monitoring and not requiring hospitalization. In a survey conducted in France, most gastroenterologists reported monitoring renal function once or twice a year for IBD patients on 5-ASA [3]. This monitoring could significantly reduce the occurrence of acute events. The risk of AKI was associated with the female sex, Crohn’s disease, older age and the presence of common comorbidities inducing renal failure. The risk of AKI was associated with a lack of monitoring.

The use of 5-ASA was not associated with the risk of AKI, as was noted in several studies [10,15]. One explanation could be that patients treated for IBDs with 5-ASA are systematically more often monitored to prevent the potential nephrotoxicity of the treatment. In our bivariate analysis, only 3% of patients receiving 5-ASA did not have monitoring versus the 17% among the patient group not receiving 5-ASA. 

The validity of our findings was supported by the use of data from an almost exhaustive national database representing 99% of the French population. The accuracy of this resource was crucial given the low AKI incidence rate in the IBD population. Most studies based on smaller samples or with a limited follow-up could not draw definitive conclusions. However, one major limitation of this database is the lack of clinical information (e.g., about disease activity and laboratory results). Hence, we could not investigate probable kidney dysfunction due to disease activity.

In this cohort study, AKI was reported after a follow-up of almost 3 years since IBD onset. In a multicentric study analyzing 151 cases of reported cases of 5-ASA–induced nephrotoxicity, the median time of kidney injury was also 3 years after starting treatment [14]. Monitoring should be scheduled regardless of treatment exposure. The involvement of gastroenterologists and patients is crucial in achieving this objective. 

In this analysis, we focused on the potential effect of monitoring regardless of the nephrotoxicity of drugs. This latter aspect would have been difficult to analyze because of the complex relationship between treatment exposure and the occurrence of AKI. Indeed, many empirical studies have concluded a no-dose effect of 5-ASA treatment on kidney events [5,20]. Considering that most patients under treatment never experienced toxicity and only sporadic cases were reported regardless of the period of exposure, ruling on the nephrotoxicity of any treatment in an explanatory analysis is difficult. Nephrotoxicity seems to be of an idiosyncratic nature [5,21].

Regular monitoring is often recommended by experts as a preventive measure against AKI in IBD. In France, a group of experts recommended monitoring at IBD diagnosis and annually for screening extra-intestinal manifestations and evaluating treatment tolerance [19,22]. However, to date, no empirical study has highlighted whether adherence to such recommendations has a favorable impact on the kidney in IBD. The results of this study could support experts’ recommendations favoring enhanced kidney monitoring in IBD.

## 5. Conclusions

The occurrence of AKI in IBD is rare but has substantial consequences for patients. Because of the lack of consensus on the etiology of this extra-digestive manifestation and whether the etiology is drugs, genetics or disease-induced nephrotoxicity, systematic monitoring could be the best alternative for protecting the renal function of IBD patients.

## Figures and Tables

**Table 1:** Characteristics of patients with inflammatory bowel disease (IBD) without and with acute kidney events.

Characteristic	Without Kidney Events (*n* = 93,670)	With Kidney Events (*n* = 693)	*p*
Follow-up, Years (SD)	5.40 (2.03)	2.95 (2.07)	
Age at cohort inclusion, years, mean (SD)	37.24 (17.23)	43.38 (20.81)	<0.0001
Male sex	44,700 (47.72)	207 (29.87)	<0.0001
IBD subtype			
Crohn’s disease	52,197 (55.32)	437 (63.06)	
Ulcerative colitis	41,473 (43.95)	256 (36.94)	
Treatment with 5-ASA	68,848 (73.5)	415 (59.8)	<0.0001
Comorbidities			
Obesity	527 (0.55)	7 (1.01)	0.1177
Diabetes	4501 (4.81)	94 (13.56)	<0.0001
Coronary diseases	786 (0.84)	17 (2.45)	<0.0001
Recent stroke	482 (0.51)	13 (1.88)	<0.0001
Dyslipidemia	3815 (4.07)	84 (12.12)	<0.0001
Hypertension	177 (0.19)	8 (1.15)	<0.0001
Peripheral arterial disease	182 (0.19)	8 (1.15)	<0.0001
Heart failure	344 (0.37)	19 (2.74)	<0.0001

Continuous variables are presented as mean and standard deviation and compared using Student’s *t*-test, whereas categorical variables are presented as *n* (%) and compared by Chi-square test. 5-ASA, 5-aminosalicylic acid. SD, standard deviation.

**Table 2:** Risk of kidney events in IBD after adjusting for baseline and time-varying sociodemographic characteristics, monitoring and comorbidities.

Covariate		IBD Cohort (*n* = 94,363)		5-ASA Use (*n* = 69,263)	
		HR (95% CI)	*p* value	HR (95% CI)	*p* value
Age		1.01 (1.01–1.01)	<0.0001	1.01 (1.00–1.01)	0.0005
Sex	Female Male (reference)	2.32 (1.97–2.73)	<0.0001	2.02 (1.64–2.49)	<0.0001
IBD phenotype	Crohn’s disease Ulcerative colitis (reference)	1.50 (1.28–1.75)	<0.0001	1.31 (1.08–1.60)	<0.0055
Lack of monitoring		3.96 (3.20–4.90)	<0.0001	2.36 (1.51–3.68)	0.0002
**Comorbidities**					
Diabetes	Yes No (reference)	2.15 (1.68–2.73)	<0.0001	1.83 (1.33–2.50)	0.0002
Recent stroke	Yes No (reference)	2.11 (1.20–3.71)	0.087	2.10 (1.03–4.30)	0.040
Dyslipidemia	Yes No (reference)	1.78 (1.37–2.32)	<0.0001	1.95 (1.41–2.70)	<0.0001
Arterial disease	Yes No (reference)	2.71 (1.32–5.56)	0.0063	3.06 (1.24–7.53)	0.0149
Heart failure	Yes No (reference)	3.23 (2.01–5.19)	<0.0001	3.31 (1.77–6.17)	0.0002
Hypertension	Yes No (reference)	2.88 (1.41–5.87)	<0.0001	/	/

HR, hazard ratio; 95% CI, 95% confidence interval.

**Table 3:** Incidence of acute kidney impairment (AKI) in IBD patients by 5-ASA use and lack of monitoring.

	Total	Acute Kidney Events	Follow-Up, Years	Person–Years	AKI Incidence (Per 1000 Person–Years)	Crude HR (95% CI) ^1^	*p* Value
Total IBD	94,363	693	5.38	507,672	1.36		
5-ASA use	69,263	415	5.75	398,262	1.04	1 (reference)	<0.0001
No 5-ASA use	25,100	278	4.86	121,986	2.27	2.4 (2.0–2.8)	
With monitoring	87,507	587	5.6	490,039	1.19	1 (reference)	<0.0001
Without monitoring	6856	106	4.4	30,166	3.51	3.7 (3.0–4.6)	

^1^ After controlling for age and sex.

## Data Availability

Data used are owned by the French National Health Insurance System (NHIS). Use is restricted to persons certified with the NHIS.

## Appendix A. International Classification of Diseases, 10th Revision (ICD-10) Codes for Comorbidity Diagnoses

**Diagnosis of Comorbidities**	**ICD-10 Codes**
Obesity	E66
Diabetes	E10–E14
Cardiovascular diseases including coronaries disease	I20–I25
Recent stroke	I63, I64
Dyslipidemia	E78
Peripheral artery disease	I702
Hypertension	I10–I15
Heart failure	I50

## Appendix B. ICD-10 Codes for Identifying Acute Kidney Events

**ICD-10 Codes**	**Designation**
N00	Acute nephritic syndrome
N01	Rapidly progressive nephritic syndrome
N04	Nephrotic syndrome
N10	Acute tubule-interstitial nephritis
N141	Nephropathy induced by other drugs, medicaments and biological substances
N142	Nephropathy induced by unspecified drug, medicament or biological substance
N144	Toxic nephropathy, not elsewhere classified
N17	Acute renal failure
